# The Italian Start Up Act: a microeconometric program evaluation

**DOI:** 10.1007/s11187-021-00468-7

**Published:** 2021-05-08

**Authors:** Francesco Biancalani, Dirk Czarnitzki, Massimo Riccaboni

**Affiliations:** 1 IMT School for Advanced Studies Lucca, Piazza S. Ponziano 6, 55100 Lucca, LU Italy; 2 Dept. of Management, Strategy and Innovation (MSI), KU Leuven, Naamsestraat 69, 3000 Leuven, Belgium; 3 Centre for R&D Monitoring (ECOOM), KU Leuven, Naamsestraat 61, 3000 Leuven, Belgium; 4 Centre for European Economic Research (ZEW), L7,1, 68161 Mannheim, Germany

**Keywords:** Startup, Innovation policy, Firm subsidies, Innovation, M13, O38, L26

## Abstract

This paper analyzes the impact of the Italian Start Up Act which entered into force in October 2012. This public policy provides a unique bundle of benefits, such as tax incentives, public loan guarantees, and a more flexible labor law, for firms registered as “innovative startups” in Italy. This legislation has been implemented by the Italian government to increase innovativeness of small and young enterprises by facilitating access to (external) capital and (high-skilled) labor. Consequently, the goal of our evaluation is to assess the impact of the policy on equity, debt, and employment. Using various conditional difference-in-difference models, we find that the Italian innovative startup policy has met its primary objectives. The econometric results strongly suggest that Italian innovative startups are more successful in obtaining equity and debt capital and they also hire more employees because of the program participation.

## Introduction

The crucial role of startups in job creation is well known and widely supported (Acs and Audretsch 1988; Davis et al. 1996; Symeonidis 1996; Freel 2005; Hausman 2005; Lee and Sung 2005; Laforet and Tann 2006; Colombo et al. 2012, Criscuolo et al. 2014; Baregheh et al. 2016, Menon et al. 2018). Empirical evidence generally confirms firm size and age to be negatively correlated with rates of job creation and firm growth (Birch 1981; Buldyrev et al. 2020; Harhoff et al. 1998; Buldyrev et al. 2007; Headd and Kirchhoff 2009; Haltiwanger et al. 2013). Moreover, it has been found that firm births account for a significant share of net job creation since firms do not grow much after an initial high growth period (Armington and Odle 1982; Kirchhoff and Phillips 1988; Audretsch and Mahmood 1994; Broersma and Gautier 1997; Voulgaris et al. 2005; Lotti 2007), and some argue that only some *small and young* firms have high growth potential, the so-called gazelles (Delmar et al. 2003; Acs and Mueller 2008).

Even though startup companies are often seen as the engine of innovation and growth, unfortunately, these companies are also known to be the most financially constrained (Himmelberg and Petersen 1994; Carpenter and Petersen 2002; Schneider and Veugelers 2010). Retrieving information on startups is more expensive for potential lenders; their securities are less frequently traded, and their financial statements do not have to be audited. Information asymmetries between insiders and external potential investors and stakeholders are magnified by the overlap of ownership and management in most of the young and small firms. The theory thus suggests asymmetric information to induce an adverse selection, about debt financing. Empirical evidence indeed confirms that the problems above cause an insufficient provision of capital to young, innovative and small firms (Audretsch and Lehmann 2004; Freel 2007; Stucki 2013; Duarte et al. 2016; Bergner et al. 2017).

The lack of assets to pledge as collateral is another problem of startups, particularly innovative newly founded firms centered around R&D activities (Hall 2002). There is a general tendency to consider R&D investments are riskier than other investments with negative consequences both for financing, as investors discount uncertainty, and for debt financing, since collateralization becomes problematic due to sunk costs and intangibles (Hall et al. 2016). Moreover, the problems of contract incompleteness and information asymmetry between firm and investors are exacerbated in the case of R&D financing (Hall and Lerner 2010). Consequently, innovative firms rely more on their own internal finance, when available. Market failures in innovation can be particularly severe in countries that lack well-functioning capital markets for innovative startups (Myers and Majluf 1984).

In order to correct these market failures, governments have established various policies. Those include R&D and innovation grant programs (see e.g. David et al. n.d., and Zúñiga-Vicente et al. 2014 for surveys) where small and young firms are often receiving a preferential treatment (see, e.g., Czarnitzki and Lopes-Bento 2013, Mas-Tur and Moya 2015), public loan guarantees (see, e.g., Ughetto et al. 2017), R&D tax credits (see, e.g., Hall and Van Reenen 2000, for a survey, or Kobayashi 2014, for evidence on Japanese SMEs), as well as public venture capital (see, e.g., Lerner 1999; Grilli and Murtinu 2015; Colombo et al. 2007) and other initiatives such as science parks (see, e.g., Wallsten 2001, Phan et al. 2005), and support for university spinoffs (Civera et al. 2020). As pointed out by Colombelli et al. (2019), young innovative companies may benefit from different policies, with a mixture of interventions that varies in time and across jurisdictions.

The purpose of this paper is to econometrically evaluate the Italian Start Up Act, which is an extensive policy framework combining a number of well-known policies to a unique bundle which may create highly favorable business conditions for innovative startups^1^. Italy, especially in the aftermath of the 2008–2009 financial crisis followed by the economic recession and the sovereign debt crisis, can be considered as one of those countries where the functioning of the financial markets for innovative startups became highly debatable, at the very least. The recognition that the crisis might have hit innovative, small and young firms more severely than other companies called for policy actions, especially for disadvantaged but potentially highly important companies for technological advancement and economic growth (cf. OECD 2009; OECD 2014; and Bergner et al. 2017; Gambardella et al. 2008).

Since small and young firms have a high demand for capital but experience problems in the acquisition of funds especially during recessions (Gompers and Lerner 2001; Audretsch and Lehmann 2004; North et al. 2013), Italy passed the Decree Law 179/2012 transformed into Law 221/2012 (the so-called Start Up Act), which can be seen as an active policy to create a more favorable environment for innovative startups. This policy scheme is a composite measure made of a set of complementary interventions aimed at unleashing the growth potential of innovative young and small firms. Among other features, it combines investment tax benefits, public loan guarantees, and a more flexible labor legislation as benefits for the program participants.

In the empirical analysis, we apply comprehensive state-of-the-art econometric techniques to estimate treatment effects of the policy on relevant target performance measures at the firm-level. We mainly rely on difference-in-difference (DiD) regressions with adequate control group designs, but also address possible self-selection mechanisms and attrition. We furthermore use our results of the treatment effects estimation to conduct a cost-benefit analysis in terms of public cost of the program and its associated results. Our analysis contributes to the debate on the motivations and objectives of policies supporting innovative entrepreneurship (see also Finaldi et al. 2016; Giraudo et al. 2019; Menon et al. 2018).

The remainder of this paper is organized as follows: Section 2 discusses the policy background, some existing literature on the Italian Start Up Act, and derives our research hypotheses. Section 3 introduces the data. Section 4 presents the empirical strategy, Section 5 shows results, and Section 6 concludes.

## Policy background, the Italian Start Up Act, and research hypotheses

### Policy background

As already experimented across the world, industrial policies to be effective must target a specific population of firms. Targeted firms can be selected according to multiple criteria such as age, size, location, industry, and R&D intensity. The Italian policy framework for innovative startups, also known as Start Up Act, is a remarkable example of the recent evolution of targeted industrial and innovative policy. The innovative startup policy was first introduced in October 2012 by the Decree Law 179, which has been transformed into Law 221/2012 in December 2012. The Start Up Act has been followed by a set of policy interventions to create a complete and coherent policy framework.

The primary objective of the Italian Law 221/2012 is “[...] to create favorable conditions for the establishment and the development of innovative enterprises to contribute significantly to economic growth and employment, especially youth employment.” (Italian Ministry of Economic Development 2016, p. 3). The Italian Law 221/2012 includes a set of support measures as listed in the “Restart, Italia!” report by the Minister of Economic Development.^2^

In addition to the main goals, this policy is also meant to contribute to filling the gap between Italy and other OECD countries regarding high-tech startups and high-skilled labor force. Italy is well-known to be the country with the most considerable fraction of micro (< 10 employees) and small firms (< 50 employees) among OECD countries. Also, small firms in Italy account for the most relevant share of total employment among OECD countries, well above 60% of total employment (Criscuolo et al. 2014). By taking a closer look at the age composition of small business, we notice that in Italy, more than one-half of small companies are older than 5 years. Young firms (i.e., firms aged less than 5 years) represent a minority of small businesses in OECD countries, but only Finland has a lower share of young firms than Italy (Criscuolo et al. 2014).^3^

Against this background, the Italian Law 221/2012 has been prompted by the Italian government to stimulate the new and young high-tech companies operational for less than 5 years employing high-skilled personnel thanks to targeted incentives to new entrepreneurial ventures. The Decree Law 179, turned into Law 221 in December 2012, introduced a dedicated policy support for innovative startups into the Italian legislation. In April 2013, another decree clarified that the access of loan guarantees is free for innovative startups. Few months later, three different decrees (Decree 30th January 2014, Decree 24th February 2016, and Decree 7th May 2019) fixed some issues on the benefits for equity investors in innovative startups (e.g., the maximum benefits, the procedure to ask for the benefits). In 2016, two decrees (Decree 17th February 2016 and Decree 28th October 2016) introduced new aspects about special format of statutes (with allowed variations) for innovative startups and an online procedure to establish new innovative startup, instead of the expensive procedure through notary. In 2018, the Italian Stock Exchange Authority clarified the use of equity crowdfunding for innovative startups through its regulation (Consob, regulation 3rd January 2018). Finally, in 2020, as exceptional measures against the COVID-19 crisis, the Italian Government inserted in Decree Law 19th May 2020 (known as Revival Decree) some temporary incentives for innovative startups.

In our analysis, we focus on the impact of the Start Up Act in the period 2012–2015. Even if the policy to boost innovative startups has changed over time, the core of the policy intervention has remained substantially unchanged until 2015. In 2015, a new policy came into force to support innovative SMEs older than 5 years (Decree Law 3/2015).

Moreover, in Italy, there is a dedicated legislative framework for university spinoffs, regulated by Decree 168/2011. University spinoffs are potentially eligible to be innovative startups or, for the ones older than 5 years, innovative small medium enterprises, but some university spinoffs are neither innovative startups nor innovative SMEs^4^. Therefore, the Start Up Act largely supported Italian university spinoffs, since recently founded university spinoffs are eligible to be registered as innovative startups. For a recent study about Italian university spinoffs, see Civera et al. (2020).

### Eligibility criteria

According to article 25 of Decree Law no. 179/2012, the eligible enterprises are small newly incorporated companies headquartered in Italy, which have been operational for less than 5 years and with a yearly turnover lower than 5 million Euros. According to the Law, innovative startups must develop and commercialize innovative products or services of high technological value. Besides, they should fulfill at least one of the following criteria^5^:

- at least 15% of the company’s expenses can be attributed to R&D activities;
- at least 1/3 of the employees are PhD students, the holders of a PhD, or researchers; alternatively, 2/3 of the total workforce must hold a Master’s degree;
- the enterprise is the holder, depositary, or licensee of a registered patent or software (intellectual property).

As only a small group of young and upcoming enterprises accounts for the bulk of net job creation, Italian Law 221/2012 targets incentives more specifically to those firms. In a nutshell, Italian Law 221/2012 is meant to mitigate financial constraints and to unleash high growth potential firms to create new qualified jobs.

Firms that meet all the criteria set by Law 221/2012 can register free of charge at a special register of “innovative startups” and are entitled to the benefits of the new legislative framework. This aspect of the policy is particularly important to evaluate the impact of the new legislation, since it rules out any risk of contamination of the treated group of firms: only registered firms get access to the benefits of the policy, with no exception. The main benefits for innovative startups can be divided into three categories:

- tax incentives for equity investments;
- a simplified procedure to get credit guarantees on bank loans; and
- tailored made labor rules to subscribe fixed-term contracts which last up to 4 years.

Investors in innovative startups get a 30% tax credit as individuals and fiscal deduction as legal entities (as of 2016). As for credit guarantees, it covers up to 80% of the bank loans and up to a maximum of 2.5 million EUR, and it is provided through a Government Fund called “Fondo Centrale di Garanzia”. When firms are no more eligible for the benefits of the policy, they exit the “innovative startup” register, and special treatments immediately stop. A report is published every year by the Italian Ministry of Economic Development, providing an in-depth analysis of the evolution of the policy, its impact, and cost^6^. Since the main interventions are on equity investments, access to bank loans, and employment, we will focus on whether this new policy has spurred equity collection, bank loans, and creation of new jobs by startup firms, conditional upon survival.

### Extant literature on the Italian Start Up Act

The Italian Start Up Act has been already investigated by a few recent published studies and ongoing works. This literature covers different aspects of the Italian Law 221/2012. Finaldi et al. (2016) analyze program participants of the years 2013 and 2014 and compare them to similar firms that did not enroll in the Innovative Start Up Act. They apply nearest neighbor propensity score matching based on covariates in the year 2012 and conduct a difference-in-differences regression in order to uncover possible treatment effects as changes in outcome variables between 2012 and 2014. However, they do not account for firm-fixed effects in their analysis. They estimated a difference-in-differences regression on a pooled cross-sectional sample of innovative startups (i.e., treated firms) and control firms; sometimes, this method is called grouped difference-in-differences (see, e.g., Wooldridge 2010). This method is rarely used in firm-level treatment effects studies as the estimates are most likely affected by unobserved heterogeneity across the two groups. Finaldi et al. (2016) find that the group of innovative startups (i.e., treated firms) shows, on average, a higher change in their bank loans than the control group and the additional capital inflow seems to be transformed into a higher investment rate, as the results of the regression analysis suggests. Even though Finaldi et al. (2016) analyze a number of other outcome variables, no further statistically significant result is found.

Menon et al. (2018) take the analysis a step further and employ a difference-in-differences regression accounting for unobserved heterogeneity across firms. Their analysis also controls for cohort-, age-, and regional-specific shocks over time. According to their results, the policy has a positive effect on a number of balance sheet variables, such as assets, book value of capital, investment, the ratio of intangible investment over tangible investment, and value added. Unfortunately, they do not give any theoretical explanation or guidance how for instance immediate target variables of the policy such as equity and debt are supposed to be transformed or related to the outcome variables that they consider in their analysis. While it is straightforward that higher equity investment and access to bank loans should increase the total assets of the company, it is not necessarily intuitive why the policy should increase for instance the ratio of intangible investment over tangible investment. Without clear formulations of hypotheses on why this should happen and how it allows to judge whether the policy is effective or successful, these results do not seem to be informative in the end. Furthermore, Menon et al. (2018) are not clear on how their control group was created. Our reading of their text suggests that they possibly used all non-treated Italian firms, and they do not make an effort to convince the reader about the validity of their control group.

A few other studies that have investigated the Italian Start Up Act have a different focus which cannot directly be interpreted as a policy evaluation in its strict sense. They do not analyze the main target variables of the policy, but related economic outcomes such as survival and venture capital (VC) backing. Guerzoni et al. (2020) explore the performance of machine learning techniques by training supervised learning algorithms to recognize innovative companies. Subsequently, they make out of sample predictions to identify firms that would have been “innovative” and thus “eligible” firms for treatment according to the definition of the Law 221/2012, the Start Up Act. Finally, they use a composite indicator for innovation derived from their models as a regressor in a survival model. The results suggest that innovative firms are more likely to survive than the rest of the sample, but the survival premium is likely to depend on location. Their study does thus utilize the program participants to obtain a measure for innovation, but the study is not a policy analysis as such. Similarly, Ferrucci et al. (2020) investigate the survival effects and growth of the public loan guarantees within the program in combination with a Young Innovative Company (YIC) indicator variable that separated such companies from a control on non-YICs. While they find positive survival and growth effects, they do not, however, account for general program participation, but only whether a YIC made use of the public loan fund.

Giraudo et al. (2019) address how the policy interacts with other financing instruments, i.e., venture capital, and they find a segmentation effect between the use of credit guarantees on bank loans and venture capitalist investments (related to tax incentives for equity). According to their results, venture capitalists prefer to invest into software companies with low asset value. Furthermore, younger startups which are relatively smaller than the average innovative startup opt for credit guarantees. Generally, VC investment is negatively associated with the probability to obtain a government-guaranteed loan.

### Research hypotheses

Our work on the Italian Start Up Act follows the tradition of rigorous, econometric policy evaluation by estimating treatment effects on the direct target variables of the policy, i.e., equity, debt, and employment. We advance the literature by a number of features. We apply difference-in-differences (DiD) regressions that control for firm-fixed effects, and also conditional DiD (CDID) where we match firms based on a propensity score of program eligibility. We provide tests for the common trend assumption, and also add a novel feature that has been totally ignored by current literature, i.e., we account for attrition. As the program is targeting newly founded high-tech or innovative companies, the results might be significantly affected by market exits. If the survival rate of firms differs considerably between innovative startups (i.e., program participants) and the control group, estimated treatment effects ignoring such sample attrition might be misleading. In addition, we also estimate heterogeneous treatment effects in two ways: (i) we calculate annual effects, as it might have taken some time after the policy has been implemented until the participating firms benefit from the treatment, and (ii) we calculate heterogenous effect for Northern/Central and Southern Italy. A priori, it is not clear where the policy could have a greater effect. On the one hand, Southern Italy is less developed in terms of infrastructure and (financial) markets and therefore the treatment effects might be especially pronounced in participating firms in that region, as they otherwise have no chance to acquire equity or debt capital and refrain from hiring workers because of the rigid labor law. On the other hand, the firms in Southern Italy might not be as economically viable as in the Northern part and potential lenders might be more conservative and skeptical such that the treatment effects of the program may not evolve as in Northern and Central Italy. We are also the first who consider post-treatment effects. The participants, namely the innovative startups, automatically drop out of the program if they, e.g., become too old or too large (in terms of sales). This means that, for example, equity investors lose their tax benefits. In this case, it could happen that investors immediately withdraw their investments in favor of other alternatives, and the firm would possibly be as constrained as before the program participation. In that case, the policy effects would be very short-lived. Finally, we conduct a cost-benefit analysis based on the treatment effects derived from our regressions and a comparison of administrative government data on program cost.

Based on the policy scheme goals and on the literature, we formulate three clear hypotheses on the expected treatment effects. If the policy is successful, we expect positive treatment effects on

- H1: Equity
  Innovative startups (i.e., firms enrolled in the program) should be able to attract more investors than non-innovative startups (i.e., in the counterfactual situation where the program does not exist), as lenders enjoy a 30% income tax break on their possible returns. This policy increases the expected profit of equity investments and makes them thus relatively more attractive compared to others, such as stock of established companies, government bonds.
- H2: Debt capital
  Innovative startups (i.e., firms participating in the program) enjoy a government guarantee up to 80% of loans obtained. This should make them a much more attractive investment opportunity for potential lenders as the credit default risk is reduced to a very large extent.
- H3: Employment
  The less restrictive labor regulation, i.e., firms can hire on temporary basis for 48 months instead of 36 months^7^ should make firms more likely to invest in human capital compared to the counterfactual situation in which this policy in absent. Another positive effect on employment could occur because the firms may have also benefitted from higher equity and debt and can thus afford more factor inputs including human capital.

While one goal of the policy is also to increase high-skilled employment in Italian small firms, it would have been desirable to have a fourth hypothesis on only high-skilled employment in addition to total employment. Such data is unfortunately not available to us. According to the Italian government, however, 26% of innovative startups register for the program by reporting that at least 1/3 of their staff are Ph.D. holders or 2/3 are Master’s degree holders; it does not seem unlikely that the share of high-skilled labor in the population of Italian small firms is also increasing if total employment in the program-participating innovative startups is increasing. They surely have a higher number of high-skilled labors than the average Italian small firm.

## Data and descriptive statistics

To evaluate the impacts of the program, we merge the participant data as published by the Ministry of Economic Development for the years 2013 to 2015 with firm-level (accounting) data from the AIDA database of Bureau van Dijk for the years 2008 to 2015.

As the policy program is focused on young and high-tech companies, we restrict our sample to firms with similar characteristics to the treated ones (i.e., innovative startups). Namely, we analyze companies which were at most 5 years old in 2013, 2014, or 2015, respectively. In addition, their revenues must have been below 5 million EUR in at least one observed year.

Moreover, we omit firms from primary sectors because of volatile output prices, highly regulated industries, or industries with a high share of publicly owned firms, such as agriculture (NACE rev. 2 A industries), quarrying and mining (NACE rev. 2 B industries), utilities and waste management industries (NACE rev. 2 D and E industries), as well as financial, bank, real estate, insurance industries^8^. Furthermore, we apply some outlier cleaning to the data to avoid that spurious results are due to potentially erroneous entries in the AIDA database. Accordingly, we remove from our sample all “small” firms with equity greater than 100,000 EUR, and bank debts more than 500,000 EUR.

Our initial sample consists of 89,834 Italian young, small enterprises including 1,569 innovative startups (program participants). As we observe firms for multiple years, the resulting unbalanced panel contains 338,289 firm-year observations.

In our sub-sample of innovative startups, the majority (almost of the 50%) belong to only two sectors: “Computer programming, consultancy and related activities” and “Scientific R&D”. However, these two industries combined account for less than 4% of the untreated companies. Table 1 shows the distribution of firms across industries in some more detail. We sort the industries according to their importance in the program, i.e., by participation frequencies.

**Table 1:** Main industries in the program and the corresponding control group

NACE 2	Treated		Untreated	
	Frequency	Percent	Frequency	Percent
62-Computer programming, consultancy, and related activities	510	32.50	2,808	3.18
72-Scientific research and development	260	16.57	408	0.46
63-Information service activities	122	7.78	1,960	2.22
71-Architectural and engineering activities; technical testing and analysis	75	4.78	2,056	2.33
26-Manufacture of computer, electronic and optical products	68	4.33	338	0.38
74-Other professional, scientific and technical activities	65	4.14	2,780	3.15
28-Manufacture of machinery and equipment n.e.c.	63	4.02	815	0.92
70-Activities of head offices; management consultancy activities	52	3.31	4,483	5.08
27-Manufacture of electrical equipment	33	2.10	490	0.56
73-Advertising and market research	30	1.91	1,456	1.65
Other industries	291	18.59	70671	80.07

By looking at the geographic composition of our sample (Table 2), we notice that about one-fourth of the innovative startups are located in the two largest urban areas of Milan and Rome. Innovative startups are mostly located in the northern part of the country.

**Table 2:** The location of treated firms, top 10 NUTS-3 regions (provinces)

Province	Treated		Untreated	
	Frequency	Percent	Frequency	Percent
Milano (North Central Italy)	264	16.83	9,000	10.20
Roma (North Central Italy)	153	9.75	14,238	16.13
Torino (North Central Italy)	105	6.69	2,602	2.95
Napoli (Southern Italy)	58	3.70	5,175	5.86
Bologna (North Central Italy)	53	3.38	1,524	1.73
Trento (North Central Italy)	51	3.25	591	0.67
Firenze (North Central Italy)	40	2.55	1,530	1.73
Bari (Southern Italy)	38	2.42	2,032	2.30
Modena (North Central Italy)	37	2.36	996	1.13
Padova (North Central Italy)	35	2.23	1,270	1.44
Total in North Central Italy	1,255	79.99	59,313	67.20
Total in Southern Italy	314	20.01	28,952	32.80

NUTS-3 regions in Italy are called provinces, and they typically take the name of the main city in the area. In Italy, the term “region” often refers to NUTS-2 regions

Our main variables of interest are equity, debt, and employment. As we will also model the participation probability and account for firm survival, we also consider some other relevant covariates, i.e., a patent dummy (data are obtained from PATSTAT), an R&D dummy, an intangibles dummy, and firm age. Table 3 shows the variable definitions and basic descriptive statistics.

**Table 3:** Variable description and descriptive statistics (338,289 firm-year observations)

Variables		Mean	Min	Max		
Equity	Total amount of equity in thousands of Euros	17.343	0	100		
Bank debt	Total amount of bank debts in thousands of Euros	25.774	0	500		
Employment	Number of employees	2.717	1	241		
Patent dummy	Dummy variable which is equal to 1 if the company applied in the current year or the previous years for at least one patent. Otherwise the dummy is 0.	0.006	0	1		
R&D dummy	Dummy variable which is equal to 1 if the company reports R&D activities for at least 1000 Euros in the current year. Otherwise the dummy is 0.	0.024	0	1		
Intangibles dummy	Dummy variable which is equal to 1 if the company reports intangibles in its balance sheet for at least 1000 Euros in the current year. Otherwise the dummy is 0.	0.687	0	1		
Age	Number of years since the foundation year.	2.382	0	7		
Survival dummy*	Dummy is equal 1 if the company does not exit the market in the corresponding year.	0.976	0	1		

Industry, province, and time dummies omitted

*The survival dummy is not reflecting the actual number of firm exit because of the unbalanced nature of the panel. Among the 89,834 firms, 9% exit the market during our sample period. The survival dummy in the table is used to model attrition in the sample period and should not be interpreted as survival rate, as it is equal to 1 in each year that the firm survives

Table 4 shows descriptive statistics of the outcome variables for the treated firms before they enter the program and afterwards. All outcome variables increase with the treatment, on average.

**Table 4:** Summary statistics of outcome variables for treated firms, before and after treatment

	Before treatment *N* = 819			After treatment *N* = 1,473		
Variable	Mean	Min	Max	Mean	Min	Max
Equity (in 1,000 EUR)	17.612	0	100	19.364	0	100
Debt (in 1,000 EUR)	10.427	0	378	26.090	0	462
Employment (headcount)	1.418	1	18	2.039	1	26

As common in treatment effects studies that utilize panel data, we would like to compare the innovative startups (i.e., program participants) before and after treatment values of outcome variables to the control group as a descriptive preview on the difference-in-differences estimates that are presented in the subsequent econometric section. Typically, the difference-in-difference estimates are visualized by simply plotting the average values of the dependent variable before and after the treatment for the program participants (i.e., before and after to be an innovative startups) and the control group in form of event-study graphs. In our situation, however, the reality of the data is more complex than the graphs that are usually presented in textbooks, because:

- instead of a law change that happens at a single point in time, our program participation is a *staggered treatment*, i.e., firms may start to enter the program from December 2012 onwards, but can also enter at any later stage in time. This implies that the treatment status does not change in a single point in time but can change in years 2013, 2014, or 2015 of our panel;
- our panel is by construction *unbalanced* as firms can enter the program only until they are maximally 5 years old. Therefore, we only consider eligible companies in the first place, i.e., firms that were founded between 2008 and 2014. Hence, any average of an outcome variable that we would compute will not be formed by the same companies in every year. Therefore, the average might vary in a not meaningful way due to entry.
- the panel becomes unbalanced due to firm exits (*attrition*) and average values of the dependent variable might therefore be misleading as described above;
- we cannot exactly time the control group accurately in comparison to the treatment group because of the staggered treatments and the various entry and exit years.

**Fig. 1:**
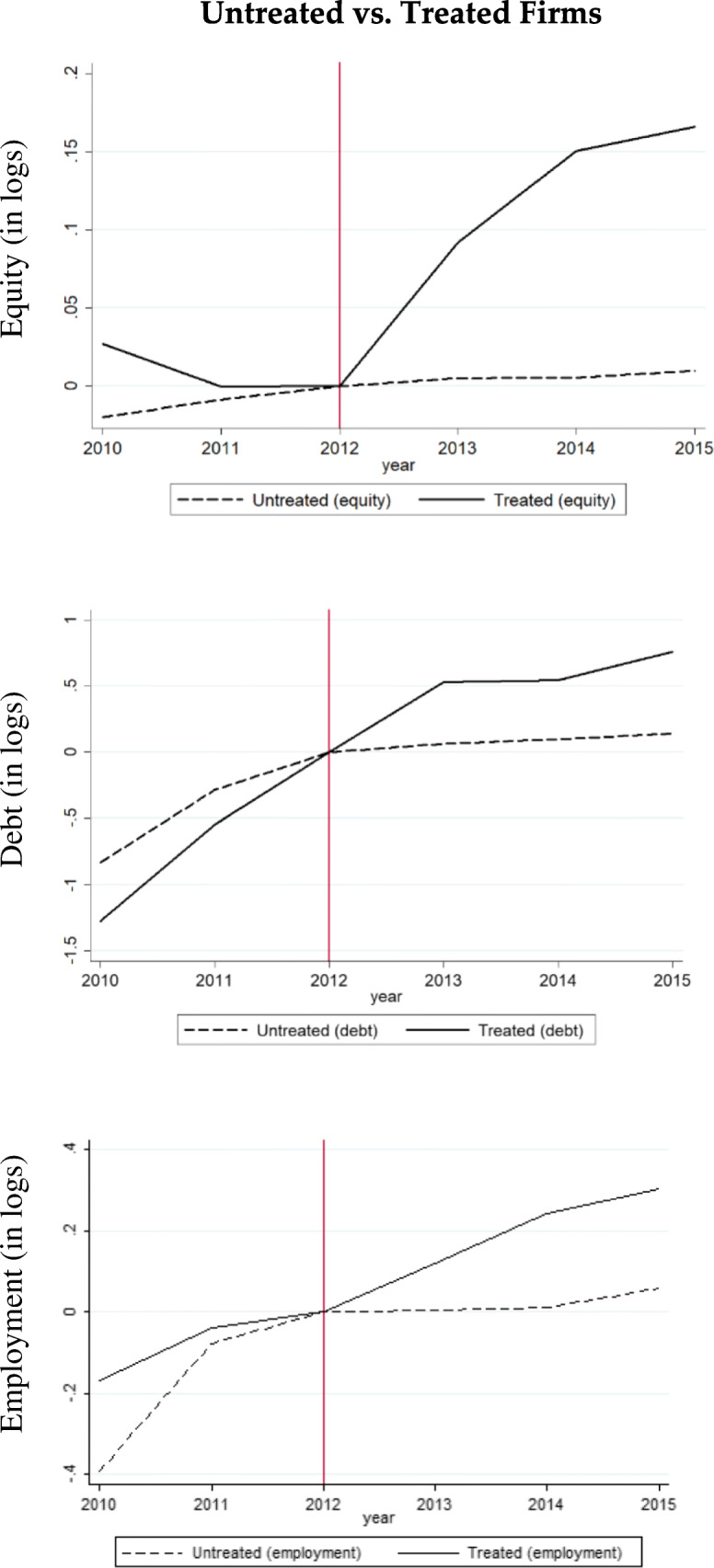
Untreated vs. treated firms. Note: The graph shows the data the foundation cohort 2010; only firms with at least 5 years of data. All firm-level data were within-demeaned, and then averaged by treatment and control group, respectively

While the subsequent regressions can be specified to account for these complexities, a simple event study graph cannot encompass all these difficulties. Therefore, we only show some graphs for the sake of illustration that simplify the real data situation. Figure 1 shows only parts of the data that is subsequently used in the regressions. In Fig. 1, only data of the foundation cohort 2010 is shown, i.e., we have two pre-treatment periods. Furthermore, the data is limited to companies for which we observe at least 5 years of data until 2015. This means we exclude early exits that make the panel unbalanced. We plot the average of logs of the dependent variables equity, debt, and employment, and rescale the mean to be zero in 2012 for each time series in order to visualize the pre-treatment trends and treatment trends as good as possible.

The graph shows a high effect in the treatment phase. The innovative startups (i.e., treated companies) show a growth of equity of about 17% between the end of 2012 and 2015, whereas the control group has a relatively flat trend only growing about 2%. In the pre-treatment period, the two groups show quite similar trends between 2011 and 2012 but the equity drops by about 3% in the treatment group between 2010 and 2011, whereas it increases by about 2.5% in the control group during the same years. Thus, it is questionable whether the common trend assumption required for valid difference-in-differences regressions hold in the data. This will be tested in the subsequent econometric specification for the whole sample by including a pre-treatment dummy variable in the regressions. When the common trend assumption turns out to be violated, we apply conditional difference-in-differences models where we match the treated firms with comparable control firms.

The same econometric methods will be applied to debt and employment. The raw data of the 2010 firm-foundation cohort shows that the innovative startups (i.e., treated companies) experienced higher growth in both the treatment period and the pre-treatment phase. Therefore, an estimate of the treatment effect without further adjustments might be misleading as the growth in debt was already higher before the Start Up Act was effective. With respect to employment, the pre-treatment trends are similar between 2011 and 2012 but the employment in untreated firms grew more between 2010 and 2011. In the treatment period, the employment of innovative startups (i.e., treated firms) grows by about 30% but only about 5% in the control group. Again, more sophisticated econometric specifications are needed for deriving a convincingly estimated treatment effect.

## Empirical strategy

For the identification of policy effects, we mainly rely on (conditional) difference-in-differences (DiD) regressions (see, e.g., Heckman et al. 1997, 1998, Angrist and Pischke 2009, 2015). We compare equity, debt, and employment of participating companies before and after the Innovative Start Up Act of December 2012. To do that, we must first identify a comparable set of non-participating firms.

The DiD estimator is usually applied to situation where a policy affects a subpopulation of companies, e.g., all small and young firms in an economy. In that case, the firms cannot self-select into treatment. It is exogenously determined which firms are in the treatment group and which firms are in the control group. In our set-up, the firms can self-select into the treatment, namely they can decide to become innovative startups. This bears some potential bias in our estimation strategy, as the firms may have different participation probabilities. For instance, there might be some firms that expect less benefits from the program than others and therefore do not select into the program. These firms may not have a growth interest in the first place and are therefore not a good control group. In order to address the self-selection problem, we also conduct the so-called conditional DiD estimations where we try to adjust the control group such that it has a similar participation likelihood as the treated firms. In that case, one would assume that the firms are either treated or not only because of purely random shocks. In practice, it means that we narrow the control group to become as similar as possible to the treatment group.

First, we consider all potentially eligible firms by size and age criteria. As tests will show, this control group does not fulfill the common trend assumption required for valid DiD estimates in all cases. Therefore, we construct more accurate control groups. As we cannot observe all other eligibility criteria comprehensively^9^, we make use of propensity score matching techniques (cf., e.g., Heckman et al. 1997, 1998) to approximate eligibility and also the participation probability to the largest extent possible. As our econometric results will show, the matched control groups conform to the common trend assumption.

In our main analysis, we select our control group according to PSM. In more detail, the probit regression used to build the propensity score considers the presence of R&D expenditures measured by an R&D dummy variable, and also the presence of intangible assets and patent applications measured by two further dummy variables.^10^ In addition, we consider the geographical location and the firms’ industry differentiated by 12 sector dummies. As the common trend assumption was not fulfilled in all DiD regressions even after matching, we also considered lagged values before the treatment period of the dependent variables as matching criteria.

As further problem specific to panel data is attrition. Attrition leads to an unbalanced panel structure due to firm exits. If firm exits are disproportional between program participants and the control group, bias may be induced in the DiD estimates. We therefore explicitly model attrition by estimating survival regressions for each year as suggested, e.g., in the textbook of Wooldridge (2010). We use the predictions of the survival regressions to compute annual, inverse Mills ratios that we include as an additional regressor in the panel DiD models.

As discussed in the literature, the standard errors in DiD applications might be biased because of autocorrelation and the so-called Moulton bias. We address this concern by clustering the standard errors at a higher level (province level) than the observational unit, as recommended in the literature (see the discussion in Bertrand et al. 2004, or Angrist and Pischke 2009).

Our first DiD specification implemented as fixed effects panel regression is:

1 $$ {\displaystyle \begin{array}{c}{y}_{it}={\gamma}_1.{\mathrm{treatment}}_{it}+{\gamma}_2.{\mathrm{post}}_{it}+{\gamma}_3.{\mathrm{before}}_{it}+\beta {X}_t+{\alpha}_i+{\varepsilon}_{it}\\ {}\mathrm{with}i=1\dots N\left(\mathrm{firms}\right)\mathrm{and}t=2008\dots 2015\left(\mathrm{years}\right)\end{array}} $$

To estimate the impact of the policy, we consider different dependent variables (*y*_*it*_): the natural logarithm of equity in thousands of Euros, the natural logarithm of bank loans in thousands of Euros, and the natural logarithm of the number of employees. Given our goal to evaluate the policy, our principal independent variable is represented by the treatment status (treatment_*it*_). We add a before treatment dummy (before_*it*_) to test whether the hypothesis of common trend assumption holds. It has value 1 the year before the treatment; otherwise, it is 0. Moreover, the post-treatment dummy (called post_*it*_) is present to avoid that formerly treated firms are considered as never-treated ones in the post-treatment phase and to estimate if the policy effects continue after the treatment period. The post-treatment dummy takes the value 1 once the firm drops out of the program because it became too large, too old, or it loses some mandatory requirements for an innovative startup (this is recorded in the administrative program data). Finally, we insert a full set of time dummies (*X*_*t*_) to control for macro-economics shocks that might affect all firms.

In addition, we propose extended specifications of our base model. As in the case of relevant attrition effects, it could happen that innovative startups (i.e., program participants) are more or less likely to survive than non-treated firms. On the one hand, innovative startups (i.e., treated firms) may be able to make riskier investment because of improved access to equity and loans. Failures of risky investment projects may increase the probability of bankruptcy and thus exit (relative to the control group). On the other hand, the improved access to capital may also allow the companies to implement their business plans appropriately which might not have been possible without the program participation. As a result, firms with well-implemented business plans might also survive longer. In order to account for attrition, we follow Wooldridge (2010: chapter 19) and estimated a series of probit regression on an indicator variable for survival. We estimate a cross-section probit model for each year *t* separately (always with the sample that was alive in *t-1*). From these probit models, we obtain the linear predictions and we calculate the inverse Mills ratio which is then included in the DiD regression as term accounting for attrition.

2 $$ {\displaystyle \begin{array}{c}{y}_{it}={\gamma}_1.{\mathrm{treatment}}_{it}+{\gamma}_2.{\mathrm{post}}_{it}+{\gamma}_3.{\mathrm{before}}_{it}+\delta .{\mathrm{Mills}}_{it}+\beta {X}_t+{\alpha}_i+{\varepsilon}_{it}\\ {}\mathrm{with}\ i=1\dots N\ \left(\mathrm{firms}\right)\ \mathrm{and}\ t=2008\dots 2015\ \left(\mathrm{years}\right).\end{array}} $$

Finally, we re-estimate Eq. (2) with matched samples constructed by PSM techniques.

## Results

### Baseline model

In this section, we show our baseline findings on the effects of the Start Up Act. Since this law provides direct incentives on collecting equity, receiving bank loans, and hiring people, we study the effects on these three variables.

As Table 5 shows, we find positive treatment effects on all three dependent variables. The equity grows about 16% in the treated firms as response to the policy. The debt increases by about 76% and employment grows by about 18%.

**Table 5:** The impact of the policy of equity, bank loans, employment: DiD regressions

Variables	(1)	(2)	(3)
	ln(equity)	ln(debt)	ln(employment)
Treatment	0.158*** (0.019)	0.758*** (0.068)	0.182*** (0.024)
Post-treatment	0.108** (0.044)	0.599*** (0.206)	0.206*** (0.058)
Before treatment	0.025* (0.014)	0.220*** (0.073)	0.047** (0.019)
Constant	2.502*** (0.005)	− 0.448*** (0.059)	0.466*** (0.017)
Firm-fixed effects	Yes	Yes	Yes
Year dummies	Yes	Yes	Yes
Observations	338,289	338,289	338,289
Number of firms	89,834	89,834	89,834

Clustered standard errors in parentheses

***p < 0.01

**p < 0.05

*p < 0.1

The post-treatment dummy is also positive and significant in all cases. For instance, in the regression on equity, it takes the value of about 11%. This would imply that the firms first manage to acquire 16% more equity as response to the policy (i.e., 16% more than they would have had if the policy would not have been introduced). Once the firm is no longer eligible to operate under the Italian Start Up Act, e.g., because it became too large or too old, investors lose their tax benefits, and as a consequence, they could withdraw their equity. The post-treatment coefficient of 10.8, however, shows that the equity remains higher than in the period before treatment. A test does not reject that the post-treatment marginal effect of 10.8 is equal to the marginal effect of the treatment dummy which is 15.8. We thus conclude that we do not find a significant withdrawal of equity after the firm has to exit the Innovative Start Up Act program. The post-treatment effects for debt and employment yield similar interpretations.

The test on common trends as indicated by the “before treatment” dummy variable is rejected in all cases, however. Thus, we conclude that these results may be affected by some bias. In order to remedy this situation, we consider further, more sophisticated estimation techniques.

### DiD models accounting for attrition

In this subsection, the DiD models account for attrition in the panel. Therefore, we estimated survival equations as suggested by Wooldridge (2010: chapter 19). These survival regressions are estimated for each year separately based on covariates of the preceding year. In the Appendix, we present a pooled cross-sectional regression for all years to save some space. The annual versions of this regression are used to compute yearly Mills ratios that are then used as an additional regressor in the DiD models to account for attrition.

Table 6 shows the DiD results for the specification accounting for attrition. The Mills ratio is negative and significant in all cases. In terms of the treatment effects, the coefficients reduce slightly. In other words, without correcting for attrition, we overestimated the effects in the initial DiD regressions. Adding the Mills ratio also reduces the coefficients and statistical significance of the “before treatment” dummy which tests the common trend assumption. However, the common trend is still rejected in the regression of debt and also weakly in the model on employment. Therefore, we turn to the matched control group below.

**Table 6:** The impact of the policy of equity, bank loans, employment: DiD regressions considering attrition

Variables	(1)	(2)	(3)
	ln(equity)	ln(debt)	ln(employment)
Treatment	0.153*** (0.019)	0.697*** (0.071)	0.162*** (0.024)
Post-treatment	0.108** (0.044)	0.596*** (0.204)	0.205*** (0.055)
Before treatment	0.022 (0.015)	0.178** (0.074)	0.033* (0.019)
Mills ratio	− 0.279*** (0.022)	− 3.701*** (0.128)	− 1.221*** (0.055)
Constant	2.499*** (0.005)	− 0.498*** (0.061)	0.449*** (0.017)
Firm-fixed effects	Yes	Yes	Yes
Year dummies	Yes	Yes	Yes
Observations	338,289	338,289	338,289
Number of firms	89,834	89,834	89,834

Clustered standard errors in parentheses

***p < 0.01

**p < 0.05

*p < 0.1

### DiD models accounting for attrition using matched control groups

In this subsection, we apply a propensity score matching technique to control for the selection into treatment by firms. Specifically, our PSM considers intangible assets, a dummy for positive R&D expenses, a patent dummy, as well as sets of dummy variables for the foundation years, the sector, and the location of the firm.

The patent dummy, the R&D dummy, and the intangible assets are used to approximate the program’s eligibility criteria to the best possible extent. Intangible assets may be seen as a proxy of the presence of innovation activities. R&D expenditures are explicitly mentioned as an eligibility criterion since it is required to have and R&D intensity of at least 15%. Being a patent applicant is linked with the criteria that required to be holder, depositary, or licensee of at least one industrial property. Unfortunately, we cannot observe the exact R&D intensity, nor unique software or other licenses, and we have no information on the qualification structure of the firm’s personnel. Even though this data may be available for the innovative startups (i.e., participant companies), but it is not available for the control group that never applied for the program.

Finally, to refine the control group even further, we also added lagged values of the outcome variables in pre-treatment periods in order to obtain common trends (if necessary)^11^.

The PSM is implemented as nearest neighbor matching with one nearest neighbor for each treated firm.

When using the propensity score matching, we obtain that the estimated coefficient of the pre-treatment dummy is statistically insignificant in all models, i.e., the common trend assumptions are not violated. Furthermore, Table 7 shows that the policy has a positive and significant impact on all three outcome variables: equity, bank loans, and employment.

**Table 7:** The impact of the policy of equity, bank loans, employment: DiD regressions with matched control groups

Variables	(1)	(2)	(3)
	ln(equity)	ln(debt)	ln(employment)
Treatment	0.105*** (0.022)	0.415*** (0.098)	0.117*** (0.029)
Post-treatment	0.043 (0.070)	0.218 (0.236)	0.100* (0.053)
Before treatment	0.007 (0.018)	0.125 (0.083)	0.014 (0.021)
Mills	− 0.542*** (0.163)	− 3.211*** (0.916)	− 0.458* (0.256)
Constant	2.712*** (0.053)	− 0.997** (0.446)	0.509*** (0.086)
Fixed effects	Yes	Yes	Yes
Year dummies	Yes	Yes	Yes
Observations	6,251	6,416	6,349
Number of firms	1,432	1,469	1,455

Clustered standard errors in parentheses

***p < 0.01

**p < 0.05

*p < 0.1

Even though the estimated treatment effects are highly statistically significant and positive, they are of moderate economic significance. One has to keep in mind that the innovative startups (i.e., program participants) are very young companies. They thus have very small factor endowments: on average, the treated companies had before the policy program existed or they participated an equity endowment of € 17,612, average debt of € 10,427 and 1.4 employees.

For equity, the estimated treatment effect amounts to a growth of 11.1% (=exp(0.105)-1). In terms of real effects, this implies that the equity grows as a result of the Italian Start Up Act from € 17,612 to € 19,549, or in other words an increase of € 1,937. The average debt increased by 51.4%, i.e., it changed from € 10,427 to € 15,790, and the average employment increased by about 1/5th of an employee (from 1.4 employees to 1.6 employees).

From the program implementation in December 2012 until the end of 2015, 5,145 firms had signed up for the program. In total, the program thus created about 926 more jobs in innovative startups, and injected almost € 38 million of capital into these firms (in terms of equity and debt).

The DiD models also contain the post-treatment dummy. This would in principle allow to investigate whether the treatment effect is durable after the participants can no longer operate under the Start Up Act. However, the post-treatment dummy becomes insignificant in our matched samples. Given these imprecise estimates tests, we never reject that the post-treatment effect is equal to the treatment effect. However, more research with more data after the program exit seems warranted to verify these preliminary results on post-treatment effects.

### DiD models with heterogeneous treatment effects

As robustness tests, we also estimated annual treatment effects. It could be that the treatment effect evolves over time as potential investors are not yet familiar with the program shortly after its introduction and this behaves more conservative in the beginning.

We create three dummy variables (*treatment2013*, *treatment2014*, *treatment2015*) to see how the policy effects unfolded over the years. As we can observe in Table 8, the treatment effects intensify year by year. In the case of debts and employment, the treatment effect in 2013 was insignificant. However, this growing trend may be due to the typical time lag needed to observe the actual impact of a new policy. For instance, in our case, firms need some months to collect equity, receive loans from banks, or hiring people, and the final outcome may not be realized immediately.

**Table 8:** DID with attrition and matched samples: annual treatment effects

	(1)	(2)	(3)
Variables	ln(equity)	ln(debt)	ln(employment)
Treatment2013	0.075*** (0.023)	0.098 (0.119)	0.031 (0.026)
Treatment2014	0.099*** (0.025)	0.281** (0.108)	0.084*** (0.032)
Treatment2015	0.149*** (0.031)	0.911*** (0.127)	0.256*** (0.041)
Post-treatment	0.052 (0.070)	0.297 (0.237)	0.124** (0.050)
Before treatment	0.005 (0.018)	0.094 (0.083)	0.006 (0.021)
Mills ratio	− 0.717*** (0.164)	− 5.295*** (0.963)	− 1.005*** (0.273)
Constant	2.708*** (0.053)	− 0.996** (0.450)	0.514*** (0.088)
Fixed effects	Yes	Yes	Yes
Year dummies	Yes	Yes	Yes
Observations	6,251	6,416	6,349
Number of firms	1,432	1,469	1,455

Clustered standard errors in parentheses

***p < 0.01

**p < 0.05

*p < 0.1

Finally, in Table 9, we analyze the impact of the policy in the Northern and Southern regions of Italy. This historical gap between the more developed Northern area and the Southern part of Italy has been widening after the Great Recession. Specifically, the Northern part has a developed economy. Conversely, the Southern part is a depressed region which often meets severe difficulties in growth, innovation, and employment because it has an undeveloped infrastructure system, weak institutions, and a fragile industrial base. To analyze the impact of the Start Up Act in the two macro-areas, we repeat the treatment analysis with a dummy variable for firms located in the Southern part of the country (called “*Mezzogiorno*,” in Italian language). While the results’ table suggests that the treatment effects vary across regions, tests on differences in coefficients between the North and the South do not yield any statistical result. We thus conclude that the policy works in both the Northern and the Southern regions of Italy.

**Table 9:** DiD with attrition and matched samples: Northern and Southern Italy

	(1)	(2)	(3)
Variables	ln(equity)	ln(debt)	ln(employment)
Treatment North	0.087*** (0.026)	0.426*** (0.100)	0.109*** (0.028)
Treatment South	0.192*** (0.069)	0.362** (0.146)	0.157** (0.070)
Post-treatment	0.040 (0.070)	0.220 (0.236)	0.099* (0.053)
Before treatment	0.007 (0.018)	0.125 (0.084)	0.014 (0.021)
Mills ratio	− 0.536*** (0.160)	− 3.214*** (0.917)	− 0.455* (0.256)
Constant	2.712*** (0.053)	− 0.997** (0.446)	0.509*** (0.087)
Fixed effects	Yes	Yes	Yes
Year dummies	Yes	Yes	Yes
Observations	6,251	6,416	6,349
Number of firms	1,432	1,469	1,455

Clustered standard errors in parentheses

***p < 0.01

**p < 0.05

*p < 0.1

## Conclusions

In our analysis, we documented that the effect of Italian Start Up Act (Law 221/2012) is positive along multiple dimensions by easing firms’ access to equity and debt capital. Specifically, tax benefits for new equity investors alleviate the problem of shortage in risk capital, since the estimated treatment effect is positive and statistically significant. The Start Up Act also contributes to access to bank loans by small and young enterprises. Following our results, we find that innovative startups have higher debt as a response to the program participation. We interpret this finding as better access to debt capital because of the public loan guarantees.

### Cost-benefit analysis

In total, our results suggest that the program had injected almost € 34 million in terms of equity and debt capital into Italian innovative startups between the end of 2012 and 2015.

In addition, the program created more than 900 additional jobs because of more flexible labor market regulations for firms operating under the Italian Start Up Act.

These benefits of the policy can be contrasted with the associated cost, i.e., forgone income tax revenues for the government as well as default loans for which the government had made guarantees towards the creditors:

- In 2013, the total costs due to lower taxes are € 5.9 million (audited value) and € 0.5 in terms of loss for guaranteed loans (estimated value by the government).
- In 2014, the total costs due to lower taxes are € 10.2 million (audited value) and 0.5 million in terms of loss for guaranteed loans (estimated value).
- For 2015, we do not have data for total cost, but inferring from new created innovative startups, we could expect a total cost in terms of forgone taxes and loss due to guaranteed loans of € 11/12 million.

This roughly corresponds to a total cost of € 29 million which would almost neutralize the capital injection of € 38 million in the total businesses. The policy thus mostly channels money that would have used for other purposes into innovative startups. As this, however, promises some further options for economic growth in the future, it still seems to be a policy with potential future benefits. In addition, more than 900 jobs were created in young innovative companies which would otherwise not have existed.

If one thus interprets the € 29 million cost for the government as a direct subsidy for the business sector, one could calculate that the creation of one job (for about 5 years at least) did cost the Italian taxpayer about € 32,000. This seems a justifiable amount for governmental job creation.

### Future research

Our results also contribute to a better understanding of the impact of other startup policies which have been recently implemented in several countries around the world, such as India (Companies Act 2013), Latvia (2016), Austria (startup program 2017), and Belgium (2017).

For future investigations, a number of questions seem highly interesting: in terms of the Italian Start Up Act, it would be interesting to investigate with more recent data how durable the estimated treatment effects are. Even though we estimated post-treatment effects, our results were somewhat inconclusive. This possibly owes to a limited number of post-treatment observations. With more recent data and thus more years elapsed after program participation, more reliable post-treatment effects could be estimated.

In addition, it would be worthwhile to explore to what extent the increased factor inputs in terms of capital and labor yield positive output effects in terms of sales or productivity growth. At time of writing this paper, the program was too recent to investigate output effects. Positive output effects require a number of successful operations after receiving additional equity and debt capital. The additional resources have to be transformed into factor inputs such as (high-skilled) labor which we partly investigated but also productive capital assets such as better machinery and lab equipment and so forth. In addition, the most promising determinant of productivity growth is undoubtedly successful research and development that eventually lead to process innovations and product innovations contributing thus (global) competitive advantages and eventually growth and profitability. As the time series on the post-policy implementation period was much too short to model such transformation processes from improved access to capital over factor inputs towards innovation and final growth, we refrained from investigating the whole value chain in this paper, and must instead leave this for future research where longer time series will be available.

Finally, it would be interesting to compare the policy design of the Italian Start Up Act to other international programs that also aim at (innovative) startup companies and to compare the effects of different policy designs. For instance, in the Italian Start Up Act, the government offers public loan guarantees up to 80% and a tax credit of 30% for returns from equity investment. It would be highly interesting to compare treatment effects when these parameters vary. For instance, one could wonder whether the treatment effects would be similar if the loan guarantees would be reduced in lieu of higher income tax breaks. The government could then reduce the social cost of the program by decreasing the cost of credit defaults in exchange for higher expected profits for private investors, thereby shifting the risk of failure from the public to private investors.

## Appendix

### Probit model on program participation

In order to select a matched control group, we employ a probit model on treatment status. We estimated cross-sectional models for each program cohort where the covariates stem from the last year before the firms may have entered the program. In particular, we consider an R&D dummy, a dummy for the presence of intangible assets, a dummy for the presence of at least one patent, the firm’s foundation year and its squared value to control for non-linearities, as well as sets of sector dummies and regional dummies. Moreover, we added pre-treatment values of the outcome variables, i.e., the logarithms of equity, debt, and employment in levels as of 2012 the year before the policy was launched. Thus, we match on the lagged levels of the outcome variables next to other exogenous covariates that determine the subsequent treatment probability. We also experimented with pre-treatment trends of the outcome variables (not shown in table).

We find that the selection criteria such as R&D, intangible assets, and the presence of at least one patent have the expected positive signs on future treatment probability and that these variables are also statistically significant (see column 3). The levels of debt and equity also show a positive sign in the regressions, but employment has a negative sign. This is to be expected as larger firms are more likely to not qualify for the Start Up Act because of the participation threshold in terms of firm size.

**Table 10:** Probit models on treatment status

Variables	(2)	(3)	(4)
ln(debt)	0.026*** (0.005)	0.034*** (0.005)	0.017*** (0.005)
ln(employment)		− 0.246*** (0.018)	− 0.254*** (0.019)
ln(equity)		0.157*** (0.010)	0.078*** (0.011)
R&D (dummy)			0.586*** (0.031)
Intangible (dummy)			0.540*** (0.029)
Patent (dummy)			1.263*** (0.050)
Age	− 0.032 (0.020)	− 0.063*** (0.020)	− 0.088*** (0.021)
Age^2^	− 0.049*** (0.004)	− 0.047*** (0.004)	− 0.039*** (0.004)
Constant	− 2.730*** (0.089)	− 2.842*** (0.094)	− 3.140*** (0.100)
Year dummies	Yes	Yes	Yes
Industry dummies	Yes	Yes	Yes
Regional dummies	Yes	Yes	Yes
Observations	185,206	185,206	185,206
Pseudo R-squared	0.310	0.323	0.372
Log likelihood	− 10708	− 10497	− 9744

Standard errors in parentheses

****p* < 0.01

***p* < 0.05

**p* < 0.1

### Survival effects and refining the control group

As explained in the main body of the text, we control for attrition in the sample. The estimates of the program treatment effects might be biased if treated firms live significantly longer or die significantly earlier than the control group. In order to control for that, we estimate a set of survival equations. In order to construct a Mills ratio as outlined in Wooldridge (2010: chapter 19), we estimate the survival models for each cross-section separately. In order to show the main regression results but save space, we print below regression results for the full sample using pooled cross-sectional data though. In the first 2 regressions, it turns out that the treated firms are more likely to survive. This effect of the treatment status disappears, however, once we control for debt, equity, and employment. This means the survival is not influenced by some (unobserved) program effect but by the fact that firms have better access to equity, employees, and debt capital, partly through their program participation.

**Table 11:** Probit models on firm survival

Variables	(1)	(2)	(3)	(4)	(5)
Treatment	0.195*** (0.049)	0.194*** (0.049)	0.063 (0.051)	0.080 (0.051)	0.014 (0.052)
Post-treatment		− 0.155 (0.180)	− 0.122 (0.180)	− 0.097 (0.182)	-0.158 (0.184)
ln(debt)			0.052*** (0.003)	0.039*** (0.003)	0.030*** (0.003)
ln(employment)				0.203*** (0.009)	0.179*** (0.009)
ln(equity)				0.030*** (0.006)	− 0.002 (0.007)
R&D (dummy)					− 0.016 (0.031)
Intangible (dummy)					0.307*** (0.011)
Patent (dummy)					− 0.075 (0.057)
Age			− 0.333*** (0.011)	− 0.350*** (0.011)	− 0.322*** (0.011)
Age^2^			0.037*** (0.002)	0.039*** (0.002)	0.039*** (0.002)
Constant	1.765*** (0.041)	1.765*** (0.041)	2.276*** (0.045)	2.009*** (0.047)	1.861*** (0.048)
Year dummies	Yes	Yes	Yes	Yes	Yes
Industry dummies	Yes	Yes	Yes	Yes	Yes
Regional dummies	Yes	Yes	Yes	Yes	Yes
Observations	335,661	335,661	335,661	335,661	335,661
Pseudo R-squared	0.0603	0.0603	0.0837	0.0916	0.102
Log likelihood	− 35473	− 35472	− 34588	− 34291	− 33897

Standard errors in parentheses

****p* < 0.01

***p* < 0.05

**p* < 0.1

### Correlation among main variables

As shown in the Table 12, the correlation among different variables is usually low; the highest one (in absolute value) is 0.29 between age and the R&D dummy which does not raise a concern about multicollinearity.

**Table 12:** Correlation among main variables

	Equity	Bank debts	Employment	Patent (dummy)	R&D (dummy)	Intangible (dummy)
Equity	1					
Bank debts	0.14	1				
Employment	0.04	0.11	1			
Patent (dummy)	0.02	0.001	− 0.01	1		
R&D (dummy)	0.07	0.06	0.05	0.02	1	
Intangible (dummy)	0.03	0.06	0.04	0.03	0.11	1
Age	0.09	0.13	0.07	0.02	− 0.29	0.06

## Footnotes

1 By “innovative startup,” we mean a company which meets the requirements of the Italian Start Up Act (the Decree Law 179/2012 transformed into Law 221/2012). Specifically, the policy targets startups (i.e., small and young companies) which are expected to become innovative firms.

2 See https://www.mise.gov.it/images/stories/documenti/startup_low_small.pdf.

3 ^1^ Also, Japan’s share of young companies is lower than in Italy, but Japanese data are only available at the establishment level; thus, no direct comparison is possible.

4 For instance, Spin-Pet SRL (established in 2009) is a university spinoff (recognized by University of Pisa) and innovative small medium enterprise; Relief SRL (established in 2019) is a university spinoff (recognized by Scuola Superiore Sant’Anna) and an innovative startup; Iuvo SRL (established in 2015) is a university spinoff (recognized by Scuola Superiore Sant’Anna Pisa) which is neither an innovative startup nor an innovative SME.

5 Other eligibility criteria are limited company, headquarter in Italy or headquarter in the EU with at least an operational branch in Italy, not listed in stock market, do not distribute profits, not be created by a merger or split-up (see Article 25 of Decree Law no. 179/2012). The Italian Ministry of Economic Development (2016) provides a description of the eligibility criteria in English.

6 See, for instance, the most recent report to the Italian Parliament, available here: https://www.mise.gov.it/images/stories/documenti/RELAZIONE_ANNUALE_2020_STARTUP.pdf .

7 A recent decree law (DL 87/2018 converted into Law 96/2018) reduces temporary position limit up to 24 months. Anyway, at the time of our study, the temporary position limit was 36 months.

8 It is worth noticing that only 1% of program participants are active in these sectors, according to a report of Italian Chambers of Commerce, 4th quarter 2015.

9 Among others, the eligibility criteria involve owning a patent or unique software or having an exclusive license, or an R&D intensity above 15% in terms of revenues, or at least 1/3rd of employees with Ph.D. degree or 2/3rds with master degree, etc. (cf. subsection 1.2 for details).

10 In case a firm is classified as an applicant, we set the patent dummy equal to 1 for the application year and all subsequent years. It is zero otherwise.

11 In this baseline, we consider only firms founded before 2013 because of at least 1year of pre-treatment is observed. Indeed, many firms established in 2013 and onwards born directly as “innovative startups”.
